# Green Pomegranate Waste Extract Modulates Macrophage Immunometabolism by Suppressing the ACLY–ME1 Axis and Promotes Pro-Resolving Macrophage Functions

**DOI:** 10.3390/biomedicines14091948

**Published:** 2026-08-29

**Authors:** Paolo Convertini, Simona Todisco, Michela Marsico, Alessandro Santarsiere, Ernesto Santoro, Antonio Evidente, Pierluigi Reveglia, Lucia Lecce, Stefano Superchi, Anna Santarsiero, Vittoria Infantino

**Affiliations:** 1 Department of Health Sciences, University of Basilicata, 85100 Potenza, Italy; paolo.convertini@gmail.com (P.C.); vittoria.infantino@unibas.it (V.I.); 2 Department of Basic and Applied Sciences, University of Basilicata, 85100 Potenza, Italy; simona.todisco@unibas.it (S.T.); alessandro.santarsiere@unibas.it (A.S.); ernesto.santoro@unibas.it (E.S.); 3 IRCCS CROB Istituto di Ricovero e Cura a Carattere Scientifico-Centro di Riferimento Oncologico della Basilicata, 85028 Rionero in Vulture, Italy; michela.marsico@crob.it; 4 Institute of Biomolecular Chemistry, National Research Council (CNR), 80078 Pozzuoli, Italy; antonio.evidente@unina.it; 5 Department of Clinical and Experimental Medicine, University of Foggia, 71122 Foggia, Italy; pierluigi.reveglia@unifg.it (P.R.); lucia.lecce@unifg.it (L.L.)

**Keywords:** immunometabolism, macrophage, pomegranate, ACLY, ME1, pro-resolving macrophage program

## Abstract

**Background:** Immunometabolic reprogramming is increasingly recognized as a fundamental driver of macrophage inflammatory activation. Although pomegranate polyphenols have been widely investigated for their anti-inflammatory activity, the underlying metabolic mechanisms remain poorly understood. Here, we investigated whether a pomegranate waste extract (PWE), obtained through a sustainable dimethyl carbonate (DMC)-based extraction process, modulates macrophage activation by targeting immunometabolic pathways. **Methods:** Human PBMC-derived macrophages stimulated with LPS and IFN-γ were treated with PWE. Inflammatory mediators, NF-κB transcription factor, histone H3 acetylation, and markers of inflammatory resolution were evaluated. Furthermore, the enzymatic activity of ATP citrate lyase (ACLY) and malic enzyme 1 (ME1) was determined. Rescue experiments with acetate, malate, and NADPH were performed to investigate the functional contribution of the ACLY–ME1 metabolic axis. **Results:** PWE significantly reduced NF-κB activation and the production of IL-1β, IL-6, TNF-α, ROS, NO•, and PGE_2_ without affecting cell viability. Mechanistically, PWE functionally suppressed ACLY and ME1, two central enzymes linking citrate metabolism to cytosolic acetyl-CoA and NADPH generation. Indeed, acetate supplementation restored PGE_2_ production and inflammatory cytokine secretion, whereas malate and NADPH rescued oxidative mediator production. PWE also lowered histone H3 acetylation, indicating that metabolic remodeling affected epigenetic regulation of inflammatory gene expression. Finally, PWE increased the expression of *CPT1A*, *SLC25A20*, Annexin A1, and *FPR2*, while enhancing IL-10 and 15-HETE secretion, consistent with activation of pro-resolving macrophage programs. **Conclusions:** These findings demonstrate that DMC-extracted PWE suppresses inflammatory macrophage activation primarily through immunometabolic modulation. By targeting the ACLY–ME1 metabolic axis, PWE limits acetyl-CoA- and NADPH-dependent inflammatory processes and fosters a shift toward a pro-resolving macrophage phenotype. Our work identifies this sustainable pomegranate waste extract as a promising modulator of macrophage immunometabolism.

## 1. Introduction

Inflammation is a complex biological response of the immune system to harmful stimuli, including pathogens, tissue injury, and metabolic disturbances [1]. Upon activation by exogenous or endogenous signals, immune cells undergo profound metabolic changes to support their functional requirements [2,3]. Immunometabolism, the dynamic interplay between immune responses and cellular metabolism, is essential for maintaining cellular homeostasis and regulating the initiation, progression, and resolution of inflammation [4,5]. Distinct immune cell phenotypes exhibit specific metabolic programs that are closely associated with their functional states and the production of key signaling molecules, such as cytokines and chemokines [6].

Although macrophages have traditionally been grouped into the pro-inflammatory M1 and anti-inflammatory M2 phenotypes, this classification is now widely recognized as an oversimplification of their functional and metabolic diversity [7,8,9]. However, the M1/M2 framework remains a model for describing the distinct metabolic programs that support the pro-inflammatory and pro-resolving functions of macrophages [10]. In activated M1 macrophages, the inflammatory response involves the secretion of different mediators, including prostaglandins, nitric oxide (NO•), and reactive oxygen species (ROS), along with pro-inflammatory cytokines such as IL-1β, IL-6, and TNF-α [11]. A central role in this response is played by the transcription factor NF-κB, whose activation helps drive macrophages towards M1 polarization through the upregulation of pro-inflammatory cytokines and metabolic enzymes [12]. This inflammatory response is sustained by a profound metabolic rewiring characterized by increased glycolysis and lipid synthesis, together with disruption of the tricarboxylic acid (TCA) cycle, reduced oxidative phosphorylation (OXPHOS), and decreased fatty acid β-oxidation [13]. These metabolic changes result from specific alterations in enzymatic activity. Among these, ATP citrate lyase (ACLY) plays a pivotal role by catalyzing the conversion of cytosolic citrate into acetyl-CoA and oxaloacetate (OAA), thereby linking glucose metabolism to lipid biosynthesis and inflammatory signaling [14]. Acetyl-CoA is subsequently utilized for *de novo* fatty acid synthesis and the production of prostaglandins and other lipid mediators involved in inflammation. Instead, OAA is converted into malate, which is then oxidatively decarboxylated by malic enzyme, generating NADPH, an essential reducing equivalent required for the synthesis of ROS and NO• [15]. Interestingly, ACLY can localize to the nucleus, where it provides acetyl-CoA for histone acetylation and other epigenetic modifications [16]. Among these effects, ACLY-derived acetyl-CoA supports NF-κB acetylation, thereby establishing an ACLY/NF-κB axis that enhances the expression levels of pro-inflammatory genes [17].

Considering this metabolic scenario, increasing attention has been directed toward bioactive natural compounds able to modulate both metabolic and epigenetic pathways.

In the last decade, pomegranate (*Punica granatum* L.) has attracted considerable scientific interest owing to its high content of bioactive phytochemicals, including punicalagin, ellagic acid, and gallic acid, and several anthocyanins [18]. These phytochemical compounds exhibit a wide range of biological activities [19], including antioxidant, anti-inflammatory, immunomodulatory [20,21], and anticancer effects [22,23].

The anti-inflammatory effects of pomegranate extract and its components were demonstrated in RAW264.7 macrophages stimulated by lipopolysaccharide (LPS), where ellagic acid, gallic acid, and punicalagin significantly reduced the production of pro-inflammatory mediators, such as NO•, PGE_2_, and IL-6 [24,25]. In addition, in LPS/interferon- γ (IFN-γ)-activated J774A.1 murine macrophage-like cells, pomegranate extract and its major component punicalagin have been shown to promote a switch from the M1 phenotype to the M2 phenotype [26]. The anti-inflammatory properties have been associated with the inhibition of the MAPK signaling pathway and the NF-κB pathway [27].

In light of these considerations, the valorization of pomegranate compounds from all parts of the fruit, including its peel by-product, which is the richest part in polyphenolic compounds, has driven research aimed at developing and optimizing new extraction methods and alternative solvents [19]. Among the alternative green solvents currently under investigation, dimethyl carbonate (DMC) is one of the most promising. Owing to its low toxicity and favorable environmental profile, it has emerged as a sustainable alternative to conventional solvents such as ethyl acetate and acetonitrile [28]. Accordingly, it has recently been widely employed in numerous green synthetic applications, including the preparation of pharmaceuticals and fragrances [29,30]. Despite the well-established versatility of this green solvent, only a limited number of examples have been reported concerning its use in the extraction and valorization of wastes [31]. Notably, it has been recently employed with excellent results for the selective extraction of oleanolic acid from grape pomace [32]. However, to the best of our knowledge, its application to pomegranate peel waste valorization has never been investigated.

Based on these considerations, we explored the effects of a DMC-extract obtained from pomegranate waste (PWE) on human LPS/IFN-γ-triggered macrophages and the metabolic adaptations that support the inflammatory response. After assessing cell viability, we first evaluated the anti-inflammatory effects of PWE in macrophages differentiated from peripheral blood mononuclear cells (PBMCs) isolated from human healthy donors and stimulated with LPS/IFN-γ by measuring the levels of inflammatory mediators, such as NO•, ROS, and PGE_2_, and of pro-inflammatory cytokines, including IL-1β, IL-6, and TNF-α. We further focused on the immunometabolism, particularly on the involvement of the citrate pathway in the downstream effects induced by PWE. To this end, we analyzed whether PWE affects the expression and activity of ACLY and ME1 that contribute to the activation and maintenance of inflammatory responses by regulating the availability of acetyl-CoA and NADPH. Furthermore, we investigated the acetylation of histone H3 and the expression of the p65 subunit of NF-κB, the major regulator of the pro-inflammatory response. Finally, we assessed pro-resolving signals and mediators, including *FPR2* and Annexin A1 expression, as well as IL-10 and 15-HETE levels. In addition, we evaluated the expression of *CPT1A* and *SLC25A20*, two key components of the mitochondrial fatty acid β-oxidation machinery, which have been associated with metabolic reprogramming toward a pro-resolving phenotype [33].

Overall, this work aims to elucidate whether pomegranate waste-derived compounds are able to reshape macrophage inflammatory responses by modulating the ACLY–ME1 metabolic axis and pro-resolving pathways.

## 2. Materials and Methods

### 2.1. Plant Material and Extraction

Pomegranate extract was prepared following a previously described procedure with slight modifications [22]. Industrial waste obtained from the pressing of the ‘Wonderful’ variety of *Punica granatum* L. was supplied by Mercorella Agricultural Company (Scanzano Jonico, Basilicata, Italy) and used as the raw material for extraction. Briefly, 250 mg of exhausted pomegranate peel was mixed with DMC at a solid-to-liquid ratio of 1:2 (*w*/*w*) and stirred at 40 °C for 120 min. The mixture was then centrifuged at 10,000 rpm for 10 min at room temperature, and the resulting supernatant was filtered through a 0.45 μm syringe filter. The filtrate was then concentrated under reduced pressure to obtain a reddish solid, hereafter referred to as pomegranate waste extract (PWE). The chemical composition of PWE, including phenolic acids and flavonoids, was determined by LC–MS/MS analysis, according to the method described in [22]. The Q1 mass, the Q3 transition, the best parameters, and the retention time are reported in Supplementary Table S1. The linearity range, curve equations, LOD, and LOQ are summarized in Supplementary Table S2.

For the biological assays, the extract was first dissolved in distilled water and subsequently diluted with the same solvent to obtain the required working concentrations.

### 2.2. Isolation of Human PBMCs and Differentiation into Macrophages

Peripheral blood mononuclear cells (PBMCs) were obtained from anonymized blood samples collected from healthy adult donors after written informed consent. All procedures complied with the principles of the Declaration of Helsinki and were approved by the local Italian Committee on Human Research (REF. TS/CEURCET/CEL n. 20230044811, 3 November 2023). Whole venous blood was collected in K2-EDTA BD vacutainer tubes (Becton, Dickinson and Company, Franklin Lakes, NJ, USA) and diluted 1:2 (*v*/*v*) with Hanks’ Balanced Salt solution (HBSS, Sigma-Aldrich, St Louis, MO, USA). PBMCs were subsequently isolated by density gradient centrifugation using Histopaque-1077 (Sigma-Aldrich, St Louis, MO, USA). Following density-gradient centrifugation, PBMCs were incubated with anti-CD14 antibody-conjugated magnetic beads (MACS^®^, Miltenyi Biotec GmbH, Bergisch Gladbach, Germany), and CD14+ monocytes were isolated according to the manufacturer’s instructions. Purified CD14+ monocytes were cultured in RPMI 1640 medium (Thermo Fisher Scientific, San Jose, CA, USA) supplemented with 10% fetal bovine serum, 2 mM L-glutamine, 100 U/mL penicillin, and 100 μg/mL streptomycin and maintained at 37 °C in a humidified atmosphere containing 5% CO_2_ in the presence of 100 ng/mL recombinant human macrophage colony-stimulating factor (M-CSF; Thermo Fisher Scientific, San Jose, CA, USA) for 3 days to promote differentiation into macrophages.

### 2.3. Cell Viability Assay

Human PBMC-derived macrophages were seeded in 96-well plates at a density of 1 × 10^4^ cells per well. After 24 h, cells were treated with increasing concentrations of PWE (2, 4, 8, 16, 32, 64, 128, and 256 μg/mL) for 72 h. The control group (O) was treated with distilled water at a final concentration corresponding to the highest solvent volume used in the treatment groups (<0.1%, *v*/*v*). To assess cell viability under the experimental conditions used for the functional assays, human PBMC-derived macrophages were exposed to LPS/IFN-γ, PWE alone (4 or 32 μg/mL), or PWE plus LPS/IFN-γ for 4 and 48 h. Cell viability was determined by the CellTiter-Glo^®^ 2.0 Cell Viability Assay (Promega, Madison, WI, USA) according to the manufacturer’s instructions. Luminescence was recorded using a GloMax^®^ Discover Microplate Reader (Promega, Madison, WI, USA).

### 2.4. Cell Treatments

Human PBMC-derived macrophages were assigned to four experimental groups: (C) vehicle-treated control; (LPS/IFN-γ) cells stimulated with 1 μg/mL lipopolysaccharide (LPS) from *Salmonella enterica* serotype Typhimurium (Sigma-Aldrich, St Louis, MO, USA) and 10 ng/mL recombinant human interferon-γ (IFN-γ; Thermo Fisher Scientific, San Jose, CA, USA); (PWE 4 + LPS/IFN-γ) cells pretreated with PWE (4 μg/mL) for 1 h before LPS/IFN-γ stimulation; and (PWE 32 + LPS/IFN-γ) cells pretreated with PWE (32 μg/mL) for 1 h before LPS/IFN-γ stimulation. Cells were stimulated with LPS and IFN-γ for up to 48 h. Incubation times varied according to the experimental endpoint and are specified in the corresponding sections describing each experiment.

### 2.5. Quantification of Cytokines

PBMC-derived macrophages (5 × 10^5^ cells) were stimulated with LPS/IFN-γ for 24 h in the presence or absence of PWE (4 or 32 μg/mL), as described above. Where indicated, cells were cotreated with 5 mM sodium acetate (Sigma-Aldrich, St Louis, MO, USA). Cell culture supernatants were collected, and the concentrations of IL-1β (KHC0011), IL-6 (KHC0061), TNF-α (BMS223-4), and IL-10 (BMS215-2) were quantified using commercially available ELISA kits (Thermo Fisher Scientific, San Jose, CA, USA) according to the manufacturer’s instructions.

### 2.6. SDS-PAGE and Western Blotting

Human macrophages (1 × 10^6^ cells) were lysed in 50 μL of ice-cold 0.1% Nonidet P-40 in PBS by three freeze–thaw cycles (−80 °C for 8 min followed by 40 °C for 4 min). Cell lysates were centrifuged at 8600× *g* for 5 min at 4 °C, and the resulting supernatants were collected for subsequent protein analysis. Protein concentration was determined using the Pierce™ Coomassie (Bradford) Protein Assay Kit (Thermo Fisher Scientific, San Jose, CA, USA). Thirty micrograms of protein were mixed with 4× Laemmli Sample Buffer (Bio-Rad Laboratories, Hercules, CA, USA), heated at 95 °C for 5 min, and separated by SDS-PAGE on 4–15% Mini-PROTEAN^®^ TGX Stain-Free™ Protein Gels (Bio-Rad Laboratories, Hercules, CA, USA). Proteins were then transferred onto nitrocellulose membranes. Membranes were blocked for 1 h at room temperature in Tris-buffered saline (TBS) containing 0.5% Tween-20 and 5% nonfat dry milk or 5% bovine serum albumin (BSA) and then incubated overnight at 4 °C with the following primary antibodies: anti-NF-κB p65 (ab16502, Abcam, Cambridge, UK), phospho-ATP citrate lyase (Ser455) (#4331, Cell Signaling Technology, Danvers, MA, USA), anti-ATP citrate lyase (ab157098, Abcam), anti-ME1 (ab97445, Abcam, Cambridge, UK), anti-histone H3 acetyl (Lys9 + Lys14 + Lys18 + Lys23 + Lys27) (ab47915, Abcam), anti-histone H3 (ab1791, Abcam, Cambridge, UK), anti-annexin A1 (ab214486, Abcam, Cambridge, UK), and anti-β-actin (ab8227, Abcam, Cambridge, UK). Membranes were subsequently incubated for 1 h at room temperature with an HRP-conjugated goat anti-rabbit IgG secondary antibody (Bio-Rad Laboratories, Hercules, CA, USA). Immunoreactive bands were visualized using WesternBright™ ECL substrate (Advansta, Menlo Park, CA, USA) and detected with a ChemiDoc™ XRS imaging system (Bio-Rad Laboratories, Hercules, CA, USA). Image acquisition and densitometric analyses were performed using Image Lab software (version 6.1, Bio-Rad Laboratories, Hercules, CA, USA).

### 2.7. Quantitative Real-Time PCR (RT-qPCR)

Total RNA was isolated from 2 × 10^6^ cells using the RNeasy Plus Mini Kit (Qiagen, Hilden, Germany) according to the manufacturer’s instructions. One microgram of purified RNA was reverse transcribed using the iScript™ cDNA Synthesis Kit (Bio-Rad Laboratories, Hercules, CA, USA) on an Applied Biosystems 2720 Thermal Cycler (Applied Biosystems, Foster City, CA, USA) as per the manufacturer’s instructions. Quantitative real-time PCR (RT-qPCR) analyses were performed in triplicate using TaqMan Gene Expression Assays (Thermo Fisher Scientific, San Jose, CA, USA) targeting human *ACLY* (Hs00982738_m1, RefSeq: NM_001096.2), mitochondrial citrate carrier (*SLC25A1*) (Hs01105608_g1, RefSeq: NM_001256534.1), malic enzyme 1 (*ME1*) (Hs00159110_m1, RefSeq: NM_002395.5), malic enzyme 2 (*ME2*) (Hs00929809_g1, RefSeq: NM_001168335.1), acetyl-CoA synthetase isoform 1 (*ACSS1*) (Hs00287264_m1, RefSeq: NM_001252675.1), acetyl-CoA synthetase isoform 2 (*ACSS2*) (Hs01122829_m1, RefSeq: NM_001076552.2), mitochondrial aspartate/glutamate carrier 1 (*SLC25A12*) (Hs00186535_m1, RefSeq: NM_003705.4), mitochondrial aspartate/glutamate carrier 2 (*SLC25A13*) (Hs01573625_m1, RefSeq: NM_001160210.1), carnitine palmitoyltransferase 1A (*CPT1A*) (Hs00912671_m1, RefSeq: NM_001031847.2), mitochondrial carrier of carnitine/acylcarnitine (*SLC25A20*) (Hs00386383_m1, RefSeq: NM_000387.5), peroxisome proliferator-activated receptor alpha *PPARα* (Hs00231882_m1, RefSeq: NM_001001928.2), formyl peptide receptor 2 *FPR2* (Hs02759175_s1, RefSeq: NM_001005738.1) and the endogenous reference gene *β-actin* (Hs01060665_g1; RefSeq: NM_001101.3). Amplification reactions were performed on a 7500 Fast Real-Time PCR System (Thermo Fisher Scientific, San Jose, CA, USA). Relative transcript abundance was determined using the comparative 2^−ΔΔCt^ method [34].

### 2.8. ACLY Phosphorylation (Cell-Based ELISA)

Phosphorylation levels of ACLY were assessed using the phospho-ATP-Citrate Lyase Cell-Based ELISA Kit (A102068; Antibodies.com, Cambridge, UK), following the manufacturer’s instructions. The assay is based on the specific detection of phospho-ACLY by a primary antibody and an HRP-conjugated secondary antibody, with TMB as a chromogenic substrate. PBMC-derived macrophages (2 × 10^4^ cells/well) were stimulated for 4 h with LPS/IFN-γ in the presence or absence of PWE and processed according to the kit protocol. Absorbance was measured at 450 nm using a GloMax^®^ Discover microplate reader (Promega, Madison, WI, USA). Values were normalized to total ATP-citrate lyase expression.

### 2.9. Enzyme Activity Assays: ACLY and ME1 Activity

PBMC-derived macrophages (1 × 10^7^ cells), treated for 4 h with LPS/IFN-γ with or without PWE, were collected, washed twice with ice-cold PBS, and lysed in 0.1% Nonidet P-40 in PBS (400 μL), as described for SDS-PAGE sample preparation (Section 2.6). ACLY and ME1 enzymatic activities were measured using 150 μg of total protein, according to previously published methods [15]. The specific ACLY and ME1 activities were normalized to protein content and expressed as a percentage of C (set at 100%).

### 2.10. ROS and NO• Detection

Intracellular reactive oxygen species (ROS) and nitric oxide (NO•) production were quantified in PBMC-derived macrophages after 24 h of LPS/IFN-γ stimulation. Where indicated, cells were pretreated for 1 h with PWE (4 or 32 μg/mL) in the presence or absence of 500 μM β-nicotinamide adenine dinucleotide phosphate (NADPH; Sigma-Aldrich, St Louis, MO, USA), alone or in combination with 5 mM sodium malate (Sigma-Aldrich). ROS and NO• levels were assessed using the fluorescent probes 6-carboxy-2′,7′-dichlorodihydrofluorescein diacetate (DCF-DA; Thermo Fisher Scientific, San Jose, CA, USA) and 4-amino-5-methylamino-2′,7′-difluorofluorescein diacetate (DAF-FM DA; Thermo Fisher Scientific, San Jose, CA, USA), respectively. Cells were harvested, washed, and resuspended in PBS (1 × 10^5^ cells/100 μL), then incubated with 10 μM DCF-DA or 2.24 μM DAF-FM DA for 30 min at 37 °C in the dark. Fluorescence was subsequently measured in triplicate using a GloMax^®^ Discover microplate reader (Promega, Madison, WI, USA).

### 2.11. PGE2 Detection

Prostaglandin E2 (PGE2) levels were quantified in PBMC-derived macrophages (5 × 10^5^ cells) after 48 h of LPS/IFN-γ stimulation. Where indicated, cells were pretreated for 1 h with PWE (4 or 32 μg/mL) in the presence or absence of 5 mM sodium acetate (Sigma-Aldrich, St Louis, MO, USA). PGE2 concentration in cell culture supernatants was measured using the DetectX^®^ Prostaglandin E2 High Sensitivity Immunoassay Kit (Arbor Assays, Ann Arbor, MI, USA), according to the manufacturer’s instructions.

### 2.12. 15-HETE Detection

15-Hydroxyeicosatetraenoic acid (15-HETE) levels were quantified in human PBMC-derived macrophages (5 × 10^5^ cells) after 48 h of LPS/IFN-γ stimulation using a commercial ELISA kit (Ab133035, Abcam, Cambridge, UK) according to the manufacturer’s instructions. Briefly, standards and samples were added to the antibody-coated microplate, followed by the sequential addition of the assay reagents, including the detection antibody and enzyme conjugate. After the recommended incubation periods and washing steps, the enzymatic reaction was initiated by adding the substrate solution and stopped after 1 h of incubation. Absorbance was measured in triplicate at 405 nm using a GloMax^®^ Discover microplate reader (Promega, Madison, WI, USA). The concentration of 15-HETE in each sample was calculated using the standard calibration curve and expressed as a fold change relative to the control group.

### 2.13. Statistical Analysis

Statistical analyses were performed using GraphPad Prism software (version 8.0.2; La Jolla, CA, USA). Results are presented as mean ± standard deviation (SD) derived from three independent experiments, each carried out in triplicate. Differences among groups were evaluated using one-way ANOVA, followed by Tukey’s multiple comparisons test. Statistical significance is indicated either by asterisks (* *p* < 0.05, ** *p* < 0.01, *** *p* < 0.001) or by different letters, where groups assigned different letters differ significantly (*p* < 0.05).

Unless otherwise stated, data represent three independent biological experiments performed using macrophages derived from three different healthy donors. Each condition was analyzed in technical triplicate, and technical replicates were averaged before statistical analysis.

## 3. Results

### 3.1. Profiling of Phenolic Compounds from Pomegranate Waste By-Products in Dimethyl Carbonate (DMC)

Phenolic compounds in the pomegranate waste extract obtained using DMC as the extraction solvent (PWE) were identified and quantified by LC-MS/MS. Metabolite identification was achieved as previously described [22]. As shown in Table 1, metabolomic analysis of PWE identified punicalagin, ellagic acid, gallic acid, (-)-epigallocatechin gallate hydrate, and phloridzin among the most abundant phenolic compounds. Ellagic acid and punicalagin were the predominant constituents of the extract and, in comparison with previous studies employing ethanol [22] as well as those reported in the literature using other extraction solvents [35], the present extract showed a markedly higher ellagic acid content and a lower punicalagin content. This finding suggests that DMC may enhance the selective recovery of ellagic acid from pomegranate waste by-products.

### 3.2. PWE Modulates Pro-Inflammatory Cytokine Production and NF-κB Expression in Human LPS/IFN-γ-Activated Macrophages

Following the metabolomic characterization of PWE, we next assessed its effects on the viability of human PBMC-derived macrophages. Cells were treated with increasing concentrations of PWE, and cell viability was assessed after 72 h. PWE reduced cell viability by approximately 30% at 256 μg/mL, with a viability of 70.62 ± 7.53% relative to untreated cells. A modest decrease in cell viability (≈10%) was also observed at 64 and 128 μg/mL, with viabilities of 93.92 ± 0.58% and 91.69 ± 2.53%, respectively (Figure S1A).

Based on the preliminary 72 h viability assessment, 32 μg/mL was selected as the highest tested concentration maintaining high cell viability (>90%). A lower concentration of 4 μg/mL, corresponding to one-eighth of 32 μg/mL, was selected because it was the lowest concentration at which an effect on inflammatory mediator production was observed in preliminary functional experiments. To exclude that the effects of PWE on inflammatory mediators were associated with reduced viability under the combined treatment conditions, cell viability was also assessed in human PBMC-derived macrophages exposed to LPS/IFN-γ, PWE alone, or PWE plus LPS/IFN-γ at 4 and 32 μg/mL for 4 and 48 h. No significant reduction in viability was observed in macrophages treated with PWE in the presence of LPS/IFN-γ compared with LPS/IFN-γ-stimulated cells (Figure S1B).

**Figure 1:**
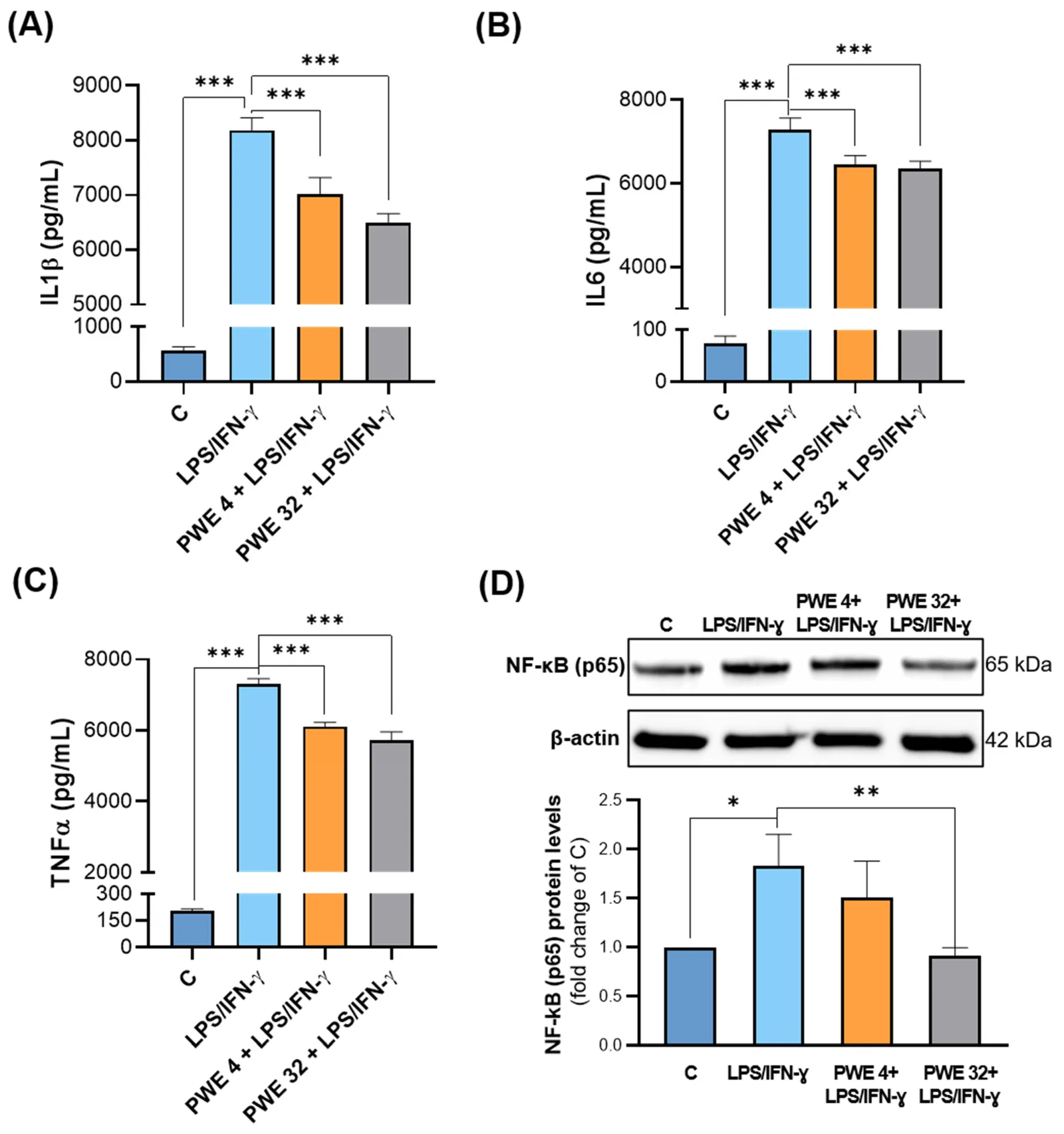
PWE attenuates the inflammatory response in LPS/IFN-γ-stimulated human macrophages. (**A**–**C**) Human PBMC-derived macrophages were stimulated with LPS/IFN-γ in the presence or absence of PWE (4 or 32 μg/mL). After 24 h of treatment, the levels of the pro-inflammatory cytokines IL-1β (**A**), IL-6 (**B**), and TNF-α (**C**) in culture supernatants were quantified. (**D**) Representative Western blot analysis of NF-κB p65 protein expression in untreated macrophages (C), LPS/IFN-γ-stimulated macrophages, and macrophages treated with LPS/IFN-γ in the presence of PWE (4 or 32 μg/mL). β-Actin was used as the loading control. Normalized densitometric analysis of NF-κB p65 protein levels from three independent experiments is shown in the bar graph below the Western blot. Data are presented as mean ± SD from three independent experiments. Statistical significance was determined by one-way ANOVA followed by Tukey’s multiple comparisons test. Statistical significance is indicated by asterisks (* *p* < 0.05, ** *p* < 0.01, *** *p* < 0.001).

The effect of PWE on the production of three proinflammatory cytokines, IL-1β, IL-6, and TNF-α, was evaluated in cell culture supernatants of LPS/IFN-γ-stimulated macrophages. Treatment with PWE at 4 and 32 μg/mL significantly reduced IL-1β, IL-6, and TNF-α levels compared with macrophages treated with LPS/IFN-γ alone (Figure 1A–C). The inhibitory activity of PWE on these pro-inflammatory cytokines was similar to that reported for pomegranate peel extract [37], confirming the anti-inflammatory properties of this waste extract obtained by DMC.

Given that the expression of pro-inflammatory cytokines is related to NF-κB, a master regulator of inflammatory responses [38], and that pomegranate extract has previously been shown to inhibit NF-κB activation [27], we investigated whether PWE also affects this pathway in human macrophages. To this end, we evaluated the protein levels of the NF-κB p65 subunit in LPS/IFN-γ-stimulated macrophages treated with PWE at 4 and 32 μg/mL. The levels of p65 were markedly reduced in cells treated with PWE at 32 μg/mL compared with cells stimulated with LPS/IFN-γ alone (Figure 1D), whereas only a non-significant reduction was observed at 4 μg/mL.

### 3.3. PWE Suppresses the Citrate Pathway in Human LPS/IFN-γ-Activated Macrophages

**Figure 2:**
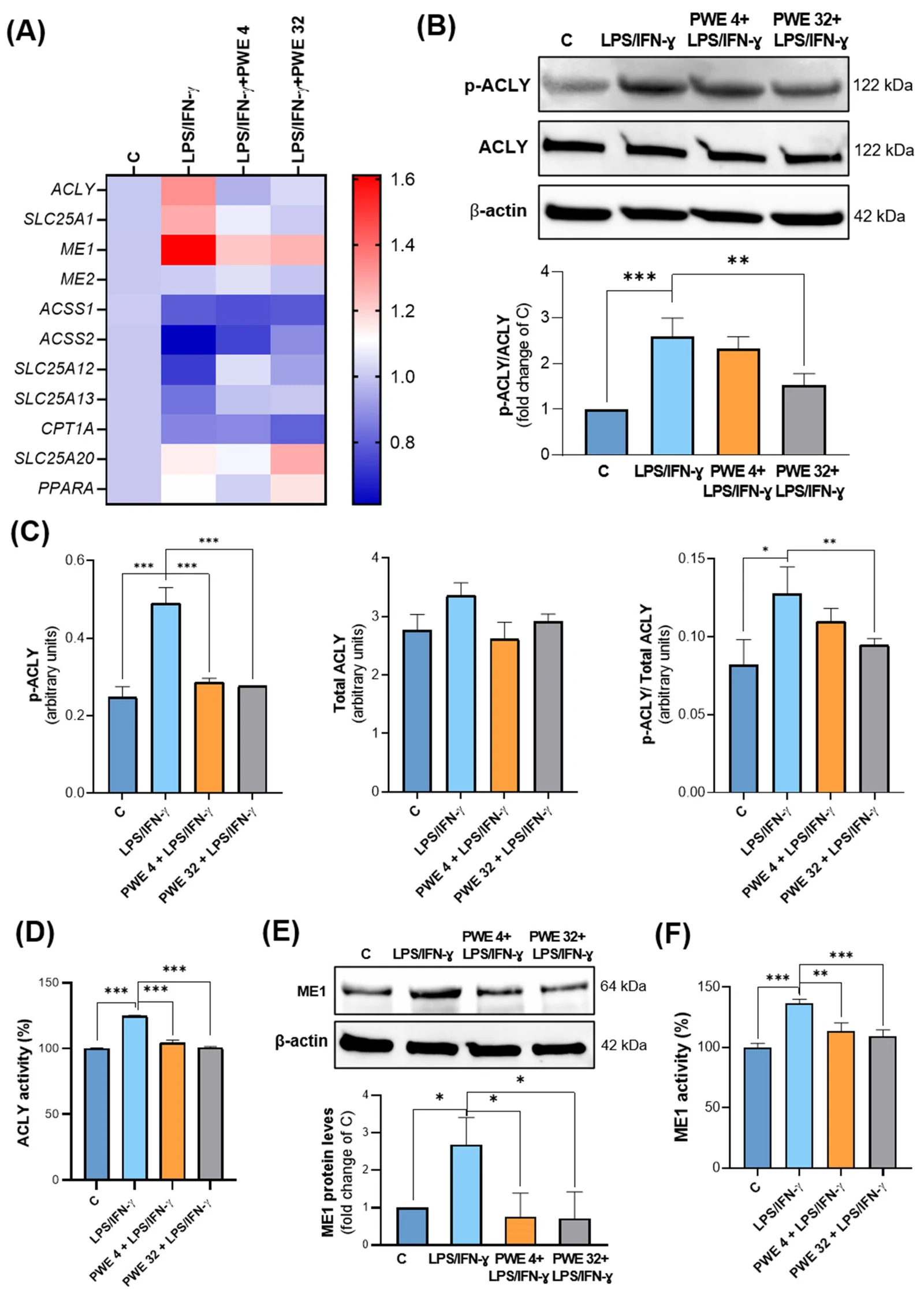
PWE suppresses the citrate pathway in LPS/IFN-γ-stimulated human macrophages. Human PBMC-derived macrophages were left untreated (C) or activated with LPS/IFN-γ for 4 h, in the presence or absence of PWE (4 or 32 μg/mL). (**A**) Heatmap showing the relative mRNA expression of genes involved in lipid metabolic reprogramming. Gene expression was determined by RT-qPCR and normalized to the β-actin housekeeping gene. (**B**) Representative Western blot of phosphorylated ACLY (Ser455) (p-ACLY), total ACLY, and β-actin. The bar graph shows the densitometric quantification of the p-ACLY/ACLY ratio from three independent experiments. (**C**) Quantification of phospho-ACLY (Ser455) (p-ACLY), total ACLY, and p-ACLY/ACLY ratio measured by ELISA. (**D**) ACLY enzymatic activity. (**E**) Representative Western blot of ME1 and β-actin. The bar graph shows the densitometric quantification of ME1 protein expression from three independent experiments, normalized to β-actin. (**F**) ME1 enzymatic activity. In (**C**,**D**,**F**) data are presented as the mean ± SD of three independent experiments. Statistical significance was determined by one-way ANOVA followed by Tukey’s multiple comparisons test. Statistical significance is indicated by asterisks (* *p* < 0.05, ** *p* < 0.01, *** *p* < 0.001).

Subsequently, the mRNA expression of genes involved in lipid metabolic reprogramming was investigated in LPS/IFN-γ-stimulated macrophages. In the early stage of activation, we observed a marked increase in the expression of *ACLY*, *SLC25A1*, and *ME1*, whereas only a slight increase was observed for the transcription factor *PPARα* and *SLC25A20* (Figure 2A, Table S3). Conversely, reduced expression was observed for *ME2*, *ACSS1* and *ACSS2*, *CPT1A*, *SLC25A12* and *SLC25A13* (Figure 2A, Table S3). Importantly, PWE treatment (4 and 32 μg/mL) significantly reversed the LPS/IFN-γ-induced increase in *ACLY*, *SLC25A1*, and *ME1* expression, suggesting that PWE could target the citrate pathway during macrophage activation. In contrast, the expression of other metabolic genes, including *ACSS1*, *ACSS2*, and *ME2*, was not significantly affected by PWE treatment, supporting a selective modulation of the ACLY–ME1 axis (Figure 2A).

To investigate the involvement of the citrate pathway, we focused on the protein expression and activity of ACLY and ME1. The protein levels of ACLY and phosphorylated ACLY (Ser455) (p-ACLY), a major activating phosphorylation site of the enzyme, increased in macrophages stimulated with LPS/IFN-γ, as determined by both Western blot (Figure 2B) and ELISA (Figure 2C) analyses. Conversely, treatment with PWE at 4 and 32 μg/mL reduced the expression of p-ACLY as well as the p-ACLY/ACLY ratio compared with cells stimulated with LPS/IFN-γ alone (Figure 2B,C). Although PWE at 4 μg/mL induced a trend toward reduced p-ACLY protein levels and a lower p-ACLY/ACLY ratio, these changes did not reach statistical significance. In contrast, treatment with PWE at 32 μg/mL significantly decreased the p-ACLY/ACLY ratio by approximately 35% and 25%, as determined by Western blot (Figure 2B) and ELISA (Figure 2C), respectively. Consistent with the increased phosphorylation of ACLY, LPS/IFN-γ stimulation significantly enhanced ACLY enzymatic activity (Figure 2D). Notably, PWE treatment completely reversed this effect, restoring ACLY activity to control levels at both 4 and 32 μg/mL (Figure 2D).

Furthermore, a similar pattern was observed for ME1. Both ME1 protein expression and enzymatic activity were significantly increased following LPS/IFN-γ stimulation. PWE treatment at both 4 and 32 μg/mL attenuated these increases, reducing ME1 protein levels and enzyme activity compared with LPS/IFN-γ-stimulated macrophages (Figure 2E,F).

### 3.4. PWE Induces Immunometabolic Rewiring in Human LPS/IFN-γ-Activated Macrophages

Given that PWE affected the expression and activity of key components of the citrate pathway, which supplies metabolites required for ROS, NO•, and PGE2 production, we next evaluated the effect of PWE on these inflammatory mediators. To further investigate the involvement of the citrate pathway, LPS/IFN-γ triggered macrophages treated with PWE 4 and 32 μg/mL were supplemented with malate, either alone or in combination with NADPH, or with acetate to replenish metabolic intermediates downstream of ACLY and ME1.

**Figure 3:**
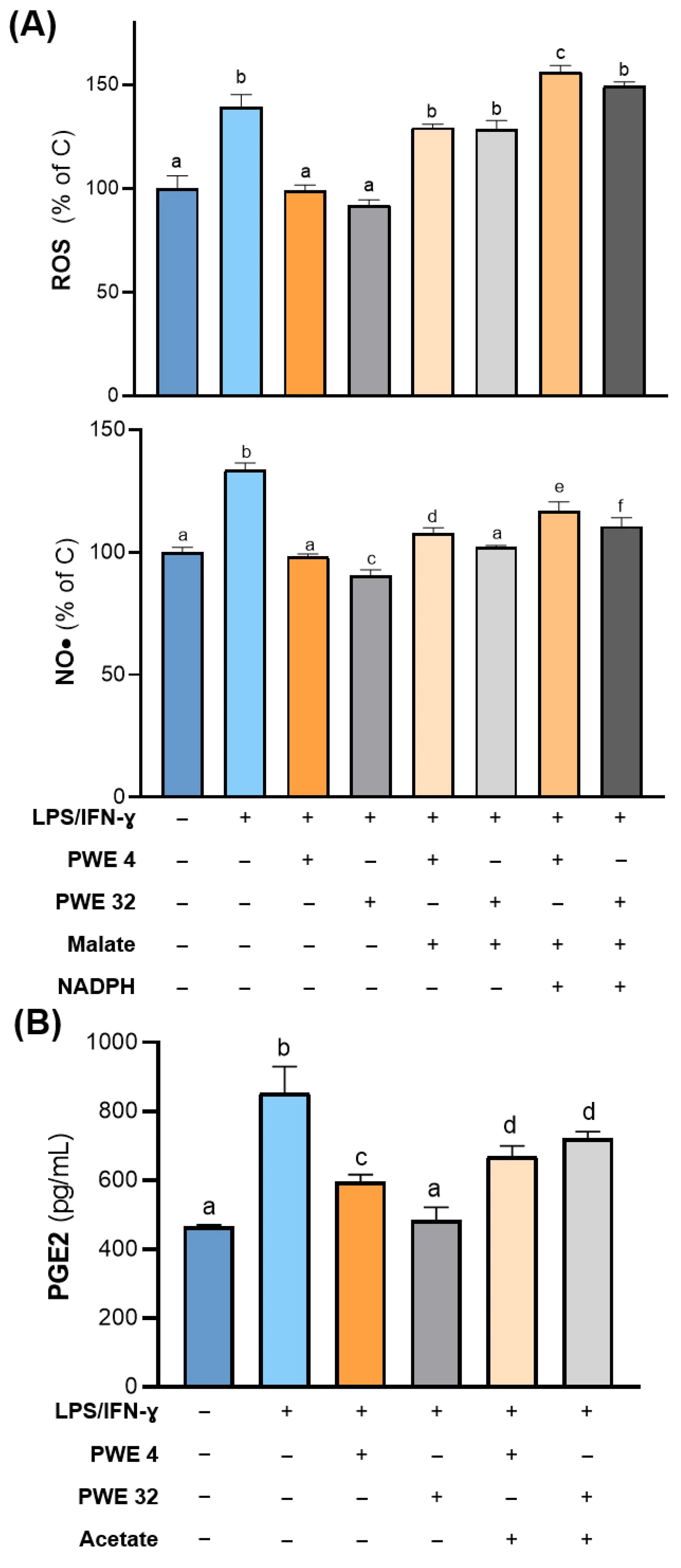
PWE modulates citrate pathway–dependent inflammatory mediator production in LPS/IFN-γ-stimulated human macrophages. (**A**) Intracellular levels of ROS and NO• in untreated macrophages (C), LPS/IFN-γ-triggered macrophages, and macrophages treated with LPS/IFN-γ in the presence of PWE (4 or 32 μg/mL), with or without supplementation of metabolic intermediates (malate and NADPH). (**B**) Production of prostaglandin E2 (PGE2) after 48 h of stimulation under the same experimental conditions, with or without supplementation of acetate. Data are presented as the mean ± SD of three independent experiments. Statistical significance was determined by one-way ANOVA followed by Tukey’s multiple comparisons test. Different letters indicate statistically significant differences.

LPS/IFN-γ stimulation markedly increased intracellular ROS and NO• levels compared with control cells. Treatment with PWE at both 4 and 32 μg/mL effectively abolished this increase, restoring ROS and NO• production to basal levels (Figure 3A). Conversely, supplementation with exogenous malate, either alone or in combination with NADPH, significantly increased ROS and NO• levels, thereby reversing the inhibitory effect of PWE (Figure 3A). These findings suggest that the reduction in ROS and NO• induced by PWE is mediated, at least in part, by suppressing the ACLY-ME1 metabolic axis.

Similar results were obtained for PGE2 production. The increase in PGE2 production observed in LPS/IFN-γ-stimulated macrophages was abolished following PWE treatment at 4 and 32 μg/mL. Notably, PGE2 levels were restored by the supplementation of acetate, which replenishes the acetyl-CoA pool broadly via ACSS2 rather than selectively mimicking ACLY-derived acetyl-CoA; this rescue supports the hypothesis that PGE2 production in activated macrophages depends on cytosolic acetyl-CoA availability (Figure 3B).

**Figure 4:**
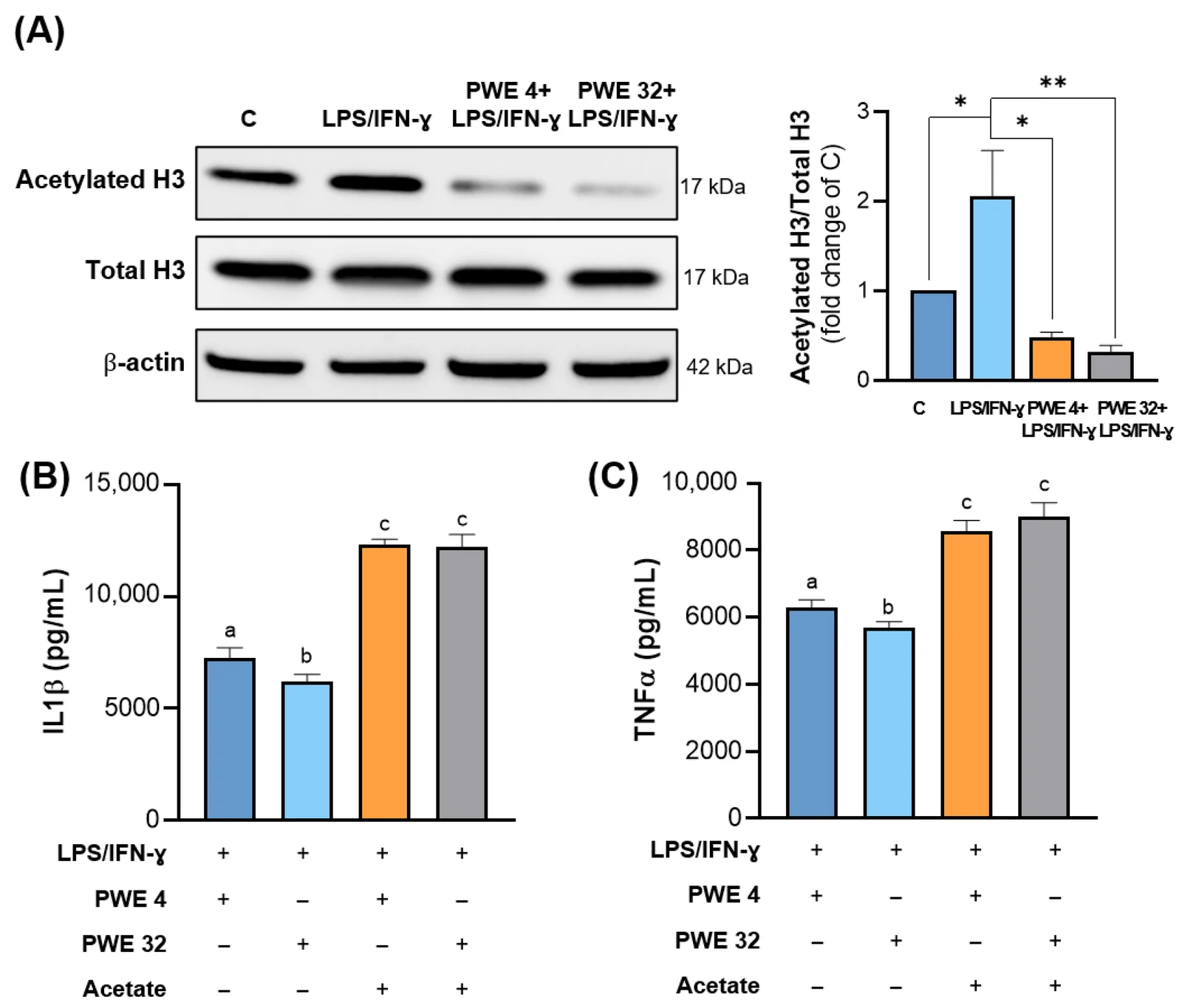
PWE reduces histone H3 acetylation, and sodium acetate rescues PWE-mediated reduction in pro-inflammatory cytokine production in LPS/IFN-γ-stimulated human macrophages. (**A**) Representative Western blot analysis of acetylated histone H3 (Ac-H3) and total histone H3 in untreated macrophages (C), LPS/IFN-γ-stimulated macrophages, and macrophages treated with LPS/IFN-γ in the presence of PWE (4 or 32 μg/mL). The bar graph shows the densitometric quantification of the acetylated H3/total H3 ratio from three independent experiments. (**B**,**C**) Levels of IL-1β (**B**) and TNF-α (**C**) in culture supernatants of PWE 4 + LPS/IFN-γ and PWE 32 + LPS/IFN-γ macrophages, with or without supplementation with sodium acetate (acetate). Data are presented as the mean ± SD of three independent experiments. Statistical significance was determined by one-way ANOVA followed by Tukey’s multiple comparisons test. In (**A**), statistical significance is indicated by asterisks (* *p* < 0.05, ** *p* < 0.01). In (**B**,**C**) different letters indicate statistically significant differences.

Based on our previous demonstration that ACLY undergoes nuclear translocation and supports NF-κB and histone H3 acetylation at pro-inflammatory gene promoters in LPS-activated human macrophages [16], we evaluated global histone H3 acetylation in the present experimental model. Acetylated histone H3 levels increased in LPS/IFN-γ-stimulated macrophages compared with control cells, whereas PWE treatment at 4 and 32 μg/mL markedly reduced global H3 acetylation (Figure 4A). In separate supplementation experiments, acetate restored IL-1β and TNF-α secretion in PWE-treated macrophages (Figure 4B,C). These findings are consistent with a potential connection between PWE-induced metabolic remodeling, acetyl-CoA availability, and epigenetic regulation.

### 3.5. PWE Promotes Pro-Resolving Reprogramming in Human LPS/IFN-γ-Activated Macrophages

**Figure 5:**
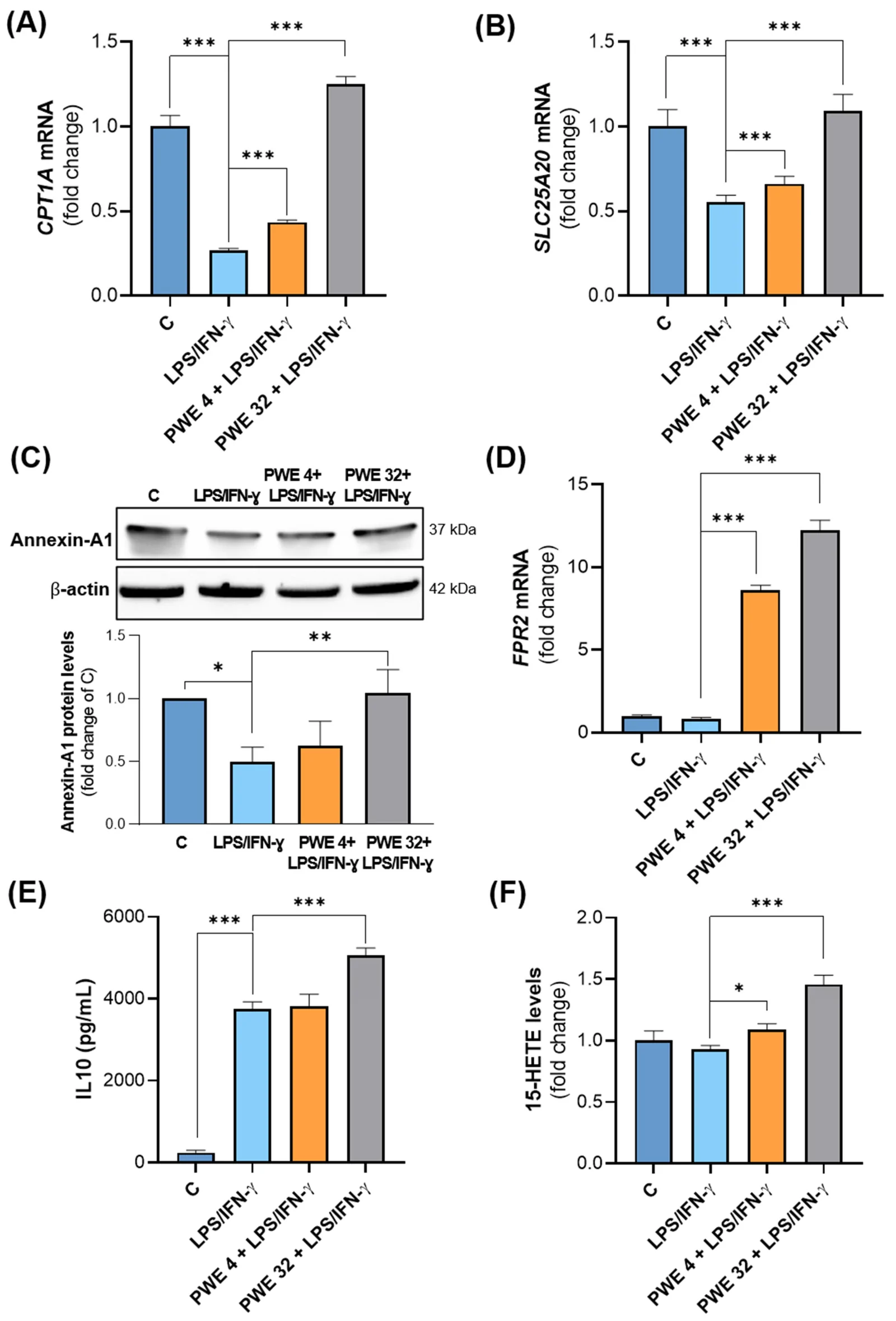
PWE promotes a pro-resolving phenotype in human LPS/IFN-γ-activated macrophages. Human macrophages were stimulated with LPS/IFN-γ for 24 h (except for *FPR2*, which was analyzed after 48 h) in the presence or absence of PWE (4 and 32 μg/mL). (**A**,**B**) Relative mRNA expression of *CPT1A* and *SLC25A20*, two key components of fatty acid β-oxidation. (**C**) Representative Western blot of Annexin A1 protein expression. The bar graph shows the densitometric quantification of Annexin A1 protein expression normalized to β-actin from three independent experiments. (**D**) Relative mRNA expression of *FPR2*. (**E**) Levels of anti-inflammatory cytokine IL-10 in cell culture supernatants. (**F**) Levels of the pro-resolving lipid mediator 15-HETE. In (**A**,**B**,**D**), target gene expression was normalized to the housekeeping gene β-actin and is expressed as fold change relative to unstimulated cells (C). Data in (**A**–**F**) are presented as the mean ± SD of three independent experiments. Statistical significance was determined by one-way ANOVA followed by Tukey’s multiple comparisons test. Statistical significance is indicated by asterisks (* *p* < 0.05, ** *p* < 0.01, *** *p* < 0.001).

To further characterize the functional impact of PWE, we evaluated whether it promotes the transition of stimulated macrophages toward an inflammatory-resolving phenotype. To address this, we first investigated the mRNA expression levels of *CPT1A* and *SLC25A20*, two enzymes involved in fatty acid β-oxidation. After 24 h of LPS/IFN-γ stimulation, *CPT1A* and *SLC25A20* mRNA levels were reduced 0.27-fold and 0.55-fold, respectively, relative to unstimulated macrophages. This temporal pattern is consistent with dynamic regulation of metabolic genes during macrophage inflammatory activation. In contrast, treatment with PWE markedly increased their expression, particularly at 32 μg/mL (Figure 5A,B). These results suggest that PWE induces metabolic rewiring in stimulated macrophages by modulating lipid metabolism.

Furthermore, among the mediators involved in the resolution of inflammation, we evaluated the expression of Annexin A1, a key pro-resolving protein that promotes a pro-reparative macrophage phenotype. As expected, LPS/IFN-γ stimulation markedly reduced Annexin A1 expression compared with untreated cells. Pretreatment with PWE at 4 μg/mL had no relevant effect, as Annexin A1 levels remained comparable to those observed in LPS/IFN-γ-stimulated macrophages. In contrast, treatment with PWE at 32 μg/mL significantly increased Annexin A1 expression, restoring protein levels to values comparable with those of untreated cells (Figure 5C).

Since Annexin A1 exerts many of its pro-resolving effects through the activation of FPR2, we next assessed *FPR2* mRNA expression. Treatment with PWE at 4 and 32 μg/mL markedly increased *FPR2* mRNA levels compared with both untreated control cells and LPS/IFN-γ-stimulated macrophages (Figure 5D).

Finally, the pro-resolving activity of PWE was further supported by the increased production of IL-10 and the elevated levels of 15-HETE observed following treatment with PWE at 32 μg/mL, compared with LPS/IFN-γ-stimulated macrophages (Figure 5E,F). At 4 μg/mL, PWE did not affect IL-10 levels, while slightly increasing 15-HETE to levels comparable to untreated cells (Figure 5E,F).

Collectively, these findings indicate that PWE not only attenuates the inflammatory response but also actively promotes the establishment of a pro-resolving macrophage phenotype. By restoring fatty acid β-oxidation and enhancing the Annexin A1/FPR2 axis together with IL-10 and 15-HETE production, PWE appears to reprogram macrophages toward a metabolic and functional state that favors the resolution of inflammation.

## 4. Discussion

Macrophage activation is increasingly recognized as a metabolically driven process in which intensive rewiring of intermediary metabolism sustains inflammatory signaling, cytokine production, and gene expression reprogramming. Within this framework, the present study demonstrates that a pomegranate waste extract (PWE) obtained using dimethyl carbonate (DMC) attenuates macrophage inflammatory activation primarily through immunometabolic reprogramming rather than by acting as a generic antioxidant.

Interestingly, the use of DMC, a sustainable solvent directly synthesized from CO_2_ [39,40], generated a phytochemical profile distinct from that obtained with conventional ethanolic extraction. Although the DMC extract contained lower amounts of punicalagins, it was enriched in ellagic acid, the principal hydrolysis product of ellagitannins. Ellagic acid is itself a bioactive polyphenol and also serves as the precursor of gut microbiota-derived urolithins, which may further contribute to the biological effects observed in vivo [18,41]. This observation is particularly interesting because it suggests that the biological properties of pomegranate extracts are determined not only by the abundance of individual polyphenols but also by the qualitative composition of the phytocomplex generated by different extraction procedures. Therefore, green extraction technologies may simultaneously improve sustainability and modulate biological activity by altering phytochemical composition.

As expected, stimulation with a combination of LPS and IFN-γ induced a classical M1-like macrophage phenotype characterized by increased NF-κB levels and enhanced secretion of IL-1β, IL-6 and TNF-α. PWE significantly weakened all these responses without affecting macrophage viability, confirming its anti-inflammatory activity. However, unlike previous studies reporting inhibition of canonical inflammatory pathways by pomegranate extracts [42,43], our data indicate that suppression of inflammatory mediators represents the downstream consequence of a broader immunometabolic reprogramming.

One of the most significant findings of this study is the identification of ATP citrate lyase (ACLY) and malic enzyme 1 (ME1) as key metabolic nodes targeted by PWE. Both enzymes are key components of the citrate–pyruvate shuttle, which links mitochondrial citrate export to cytosolic acetyl-CoA and NADPH production [16]. Accordingly, the coordinated suppression of ACLY and ME1 suggests that PWE targets a central metabolic pathway required for inflammatory macrophage functions.

Importantly, our rescue experiments strongly support this mechanistic interpretation. Supplementation with malate and NADPH substantially restored ROS and nitric oxide production in PWE-treated macrophages, whereas acetate supplementation replenished the acetyl-CoA pool, thereby rescuing PGE_2_ biosynthesis in this condition.

Acetate can provide an ACLY-independent route for acetyl-CoA synthesis through acetyl-CoA synthetases. Although *ACSS1* and *ACSS2* transcripts were reduced at the early time point examined, residual ACSS activity may still enable exogenous acetate to contribute to acetyl-CoA synthesis at later experimental time points. Accordingly, the reduction in ROS following PWE treatment should not be interpreted solely as evidence of direct antioxidant activity but may also be related to impaired NADPH-dependent ROS production associated with functional suppression of the ACLY–ME1 metabolic network.

This distinction is conceptually important. While antioxidant and metabolic mechanisms are not mutually exclusive and may both contribute to the observed effects, our results highlight a predominant role of immunometabolic rewiring in limiting the availability of intermediates required for the inflammatory program. By reducing acetyl-CoA and NADPH availability, PWE simultaneously limits prostaglandin synthesis, oxidative mediator production, nitric oxide generation and inflammatory gene expression, thereby impairing the metabolic support that fuels macrophage activation. This interpretation suggests a potential role for metabolic reprogramming upstream of inflammatory suppression, extending the effects of dietary polyphenols beyond the classical antioxidant framework [44,45].

The reduction in global histone H3 acetylation is consistent with our previous findings in human PBMC-derived macrophages, in which inflammatory activation induced ACLY nuclear translocation and increased nuclear ACLY activity. In that study, pharmacological ACLY inhibition reduced NF-κB p65 acetylation and histone H3 acetylation at the IL1B and PTGS2 promoters, thereby suppressing pro-inflammatory gene expression [17]. On this basis, the present results suggest that PWE-mediated suppression of ACLY may affect the previously characterized metabolic–epigenetic axis. However, the current study did not measure nuclear acetyl-CoA, ACLY nuclear localization, or locus-specific histone acetylation, and histone acetylation was not evaluated following acetate supplementation. Therefore, although our findings are consistent with the previously demonstrated mechanism, its specific involvement in the response to PWE remains to be directly established.

A further strength of the present study is that PWE-induced immunometabolic reprogramming coincides with activation of inflammatory resolution pathways. Resolution is now recognized as an active biological program requiring specific metabolic adaptations rather than a passive decline of inflammation [46,47,48]. In this regard, the increased expression of *CPT1A* and *SLC25A20* suggests enhanced mitochondrial fatty acid oxidation, a hallmark of macrophages acquiring reparative and pro-resolving functions [49]. Moreover, increased IL-10 secretion together with enhanced Annexin A1 expression and *FPR2* transcription indicates activation of endogenous pro-resolving circuits that promote restoration of tissue homeostasis [50]. These findings significantly advance the current understanding of pomegranate-derived phytochemicals, demonstrating that PWE suppresses inflammatory activation while simultaneously promoting a pro-resolving macrophage phenotype. However, these findings do not demonstrate direct suppression of ACLY by a specific PWE constituent. The observed activity may also result from additive or synergistic interactions among multiple components. Comparative studies using isolated compounds and defined combinations will be required to identify the constituent or phytochemical profile responsible for the observed immunometabolic effects.

Moreover, the reduction in ACLY Ser455 phosphorylation may reflect modulation of upstream signaling pathways, including AKT, which was not investigated in the present study. Further studies using different macrophage-activating stimuli and in vivo models are required to assess the potential applicability of PWE in chronic inflammatory diseases.

## 5. Conclusions

Overall, our study supports a model in which a pomegranate waste DMC-extract reshapes macrophage immunometabolism through functional suppression of the ACLY–ME1 metabolic axis. The consequent reduction in acetyl-CoA and NADPH availability disrupts the metabolic infrastructure required to sustain inflammatory activation while promoting metabolic and molecular programs associated with inflammatory resolution. Rather than acting primarily as an antioxidant, PWE appears to redirect macrophage metabolism from an inflammatory toward a pro-resolving state. These findings provide mechanistic insight into the biological activity of sustainable pomegranate waste extracts. However, although PWE increased several metabolic and molecular markers associated with inflammatory resolution, these findings should not be interpreted as evidence of M2 polarization.

## Figures and Tables

**Table 1:** Metabolomic profile of pomegranate waste extract in DMC.

Compound	DMC (mg/g)
Punicalagin	6.64 ± 0.02
Baicalein [36]	0.04 ± 0.02
(-)-Epigallocatechin gallate hydrate	0.33 ± 0.005
Apigenin	0.06 ± 0.001
Phloridzin	0.38 ± 0.02
Ellagic acid dihydrate	5.34 ± 0.06
Gallic acid	1.24 ± 0.04
Hesperidin	<LOQ *

* LOQ: limit of quantification.

## Data Availability

The original contributions presented in this study are included in the article/Supplementary Materials. Further inquiries can be directed to the corresponding authors.

## Supplementary Materials

The following supporting information can be downloaded at https://www.mdpi.com/article/10.3390/biomedicines14091948/s1, Table S1. Optimized Q1 mass, product ion and parameters for sMRM experiment of Phenolic Compounds; Table S2. Linearity and sensitivity data; Figure S1. Effect of PWE on human macrophage viability under basal and LPS/IFN-γ-stimulated conditions; Table S3. Fold-change values for genes shown in the heatmap in Figure 2A.

## Abbreviations

The following abbreviations are used in this manuscript:

Ac-H3	Acetylated Histone H3
ACLY	ATP citrate lyase
ACSS1	Acetyl-CoA synthetase short-chain family member 1
ACSS2	Acetyl-CoA synthetase short-chain family member 2
BSA	Bovine serum albumin
CO_2_	Carbon dioxide
CPT1A	Carnitine palmitoyltransferase 1A
DAF-FM DA	4-amino-5-methylamino-2′,7′-difluorofluorescein diacetate
DCF-DA	6-carboxy-2′,7′-dichlorodihydrofluorescein diacetate
DMC	Dimethyl carbonate
ELISA	Enzyme-linked immunoassay
FBS	Fetal bovine serum
FPR2	Formyl peptide receptor 2
HBSS	Hanks’ Balanced Salt Solution
HRP	Horseradish peroxidase
IFN-γ	Interferon gamma
IL-1β	Interleukin-1 beta
IL-6	Interleukin-6
IL-10	Interleukin-10
LC-MS/MS	Liquid chromatography–tandem mass spectrometry
LOD	Limit of detection
LOQ	Limit of quantification
LPS	Lipopolysaccharide
MAPK	Mitogen-activated protein kinase
M-CSF	Macrophage colony-stimulating factor
ME1	Malic enzyme 1
ME2	Malic enzyme 2
NADPH	Nicotinamide adenine dinucleotide phosphate (reduced form)
NF-κB	Nuclear factor kappa B
NO•	Nitric oxide
OAA	Oxaloacetate
OXPHOS	Oxidative phosphorylation
PBMC	Peripheral blood mononuclear cell
PBS	Phosphate-buffered saline
PGE_2_	Prostaglandin E_2_
PPARα	Peroxisome proliferator-activated receptor alpha
PWE	Pomegranate waste extract
ROS	Reactive oxygen species
RPMI	Roswell Park Memorial Institute medium
RT-qPCR	Reverse transcription quantitative polymerase chain reaction
SD	Standard deviation
SLC25A1	Solute carrier family 25 member 1
SLC25A12	Solute carrier family 25 member 12
SLC25A13	Solute carrier family 25 member 13
SLC25A20	Solute carrier family 25 member 20
TBS	Tris-buffered saline
TCA	Tricarboxylic acid
TMB	3,3′,5,5′-Tetramethylbenzidine
TNF-α	Tumor necrosis factor alpha
15-HETE	15-Hydroxyeicosatetraenoic acid
